# Large Language Model‐Based Localization of Premature Ventricular Contraction Origins: A Retrospective Diagnostic Accuracy Study

**DOI:** 10.1111/jce.70464

**Published:** 2026-08-01

**Authors:** Kazuhisa Matsumoto, Yuya Fujisaki, Syunta Higuchi, Masataka Narita, Wataru Sasaki, Tsukasa Naganuma, Naomichi Tanaka, Daisuke Kawano, Kei Matsumoto, Wataru Yoshino, Yoshifumi Ikeda, Ritsushi Kato, Hitoshi Mori

**Affiliations:** ^1^ Department of Cardiology Saitama Medical University, International Medical Center Hidaka Saitama Japan; ^2^ Sera, Inc Meguro Tokyo Japan; ^3^ Department of Clinical Engineer Saitama Medical University International Medical Center Hidaka Saitama Japan

**Keywords:** artificial intelligence, deep learning, electrocardiogram, large language model, premature ventricular contraction

## Abstract

**Background:**

Accurate localization of premature ventricular contraction (PVC) origin from 12‐lead electrocardiography (ECG) is important for procedural planning in catheter ablation. Although convolutional neural network (CNN)‐based models have shown promising diagnostic performance, they require task‐specific training and remain limited in interpretability. We evaluated whether large language model (LLM)‐based ECG image interpretation could perform binary left‐versus‐right PVC origin localization from 12‐lead ECG images while providing a traceable diagnostic process.

**Methods:**

We retrospectively studied 157 patients who underwent successful catheter ablation of PVCs or idiopathic ventricular tachycardia. ECG images were classified as RIGHT‐origin (*n* = 103) or LEFT‐origin (*n* = 54) according to the final successful ablation site. Three approaches were compared: a CNN‐based baseline model, an LLM‐based one‐shot approach, and an LLM‐based staged extraction framework with deterministic rule‐based integration. Performance was evaluated across five independent seeds using positive predictive value (PPV), negative predictive value (NPV), recall, and PPV + NPV.

**Results:**

The CNN‐based baseline model achieved a PPV of 0.546 ± 0.058 and an NPV of 0.793 ± 0.047, with area under the curve values ranging from 0.595 to 0.730. The staged extraction framework generated a continuous rule‐based score with discrimination numerically comparable to the CNN‐based model (AUC 0.720 ± 0.045 vs. 0.712 ± 0.054). A stricter threshold, determined from training data, increased PPV at the expense of recall and warrants prospective external validation of this operating point.

**Conclusions:**

LLM‐based staged extraction demonstrated the potential to achieve binary left‐versus‐right PVC origin localization from 12‐lead ECG images while providing a traceable stepwise diagnostic process. The continuous rule‐based score showed discrimination numerically comparable to the CNN‐based model, and the strict threshold—determined using training data—identified a potential high‐PPV operating point that requires prospective external validation.

**Abbreviations**
AI: artificial intelligence
AUC: area under the curve
CNN: convolutional neural network
ECG: electrocardiogram
Grad‐CAM: gradient‐weighted class activation mapping
JPEG: joint photographic experts group
LLM: large language model
LV: left ventricle
LVOT: left ventricular outflow tract
NPV: negative predictive value
OT‐PVC: outflow tract premature ventricular contraction
PPV: positive predictive value
PVC: premature ventricular contraction
ROC: receiver operating characteristic
RV: right ventricle
RVOT: right ventricular outflow tract
SSL: self‐supervised learning
VT: ventricular tachycardia

## 1 Background

Premature ventricular contractions (PVCs) are a common arrhythmia; however, when present at a high burden, they can lead to tachycardia‐induced cardiomyopathy and may trigger life‐threatening arrhythmias such as ventricular fibrillation, thereby requiring appropriate therapeutic intervention [1, 2]. Catheter ablation is an important treatment option for PVCs and can achieve high curative rates depending on the site of origin [3]. Because catheter ablation for ventricular arrhythmias originating from the left‐sided structures carries a higher risk of procedural complications compared to those from the right side [4], preprocedural localization to differentiate left‐ versus right‐sided origins is critical for clinical decision‐making and determining appropriate treatment indications. Although experienced clinicians can often infer the origin based on characteristic ECG findings, detailed morphological interpretation remains challenging for non‐specialists, representing a clinical limitation.

Recent advances in artificial intelligence (AI) have led to increasing applications in cardiovascular medicine [5, 6, 7, 8, 9, 10, 11]. In particular, deep learning approaches using convolutional neural networks (CNNs) have demonstrated high diagnostic performance [6], with reports showing approximately 90% accuracy in identifying outflow tract PVCs (OT‐PVCs) from ECG images [12, 13]. However, CNN‐based models are susceptible to overfitting when trained on limited data sets and have inherent limitations in explainability, as their decision‐making processes are not always readily interpretable despite visualization techniques such as Gradient‐weighted Class Activation Mapping (Grad‐CAM) [14]. In contrast, large language models (LLMs), such as ChatGPT (OpenAI, San Francisco, CA, USA) and Gemini (Google LLC, Mountain View, CA, USA), have rapidly emerged as a novel approach capable of probabilistic reasoning based on structured textual input without requiring additional model training [15, 16]. Unlike conventional CNN‐based methods, LLMs can provide diagnostic support through structured [11], stepwise reasoning processes, potentially overcoming some of the limitations of CNN‐based approaches. However, there are a few reports regarding the analysis of ECG images using LLMs [17, 18].

In this study, we investigated a novel method for localizing PVC origins (right‐ vs. left‐sided) by applying a structured prompt–based LLM approach on ECG image data, comparing the CNN‐based approaches.

## 2 Methods

### 2.1 Study Population

A total of 181 consecutive Japanese patients who underwent catheter ablation for PVCs or idiopathic ventricular tachycardia (VT) were initially screened. Patients treated between April 1, 2007, and August 31, 2025, were evaluated, and the indications for ablation were limited to symptomatic PVCs and VT in accordance with clinical guidelines [1]. Among them, patients in whom the site of origin was successfully identified via electro‐anatomical mapping and treated with radiofrequency catheter ablation were considered eligible. Cases were excluded based on the following criteria: (1) lack of a baseline 12‐lead ECG recording of the index PVC waveform in VT cases; (2) presence of pacemaker‐mediated rhythms; (3) verapamil‐sensitive left ventricular tachycardia with a narrow QRS morphology; and (4) presence of multifocal PVC waveforms on the 12‐lead ECG.

This study was conducted in accordance with the principles of the Declaration of Helsinki and applicable local regulations. Informed consent was obtained using an opt‐out format. The study protocol was reviewed and approved by the Hospital Institutional Review Board of Saitama Medical University International Medical Center (approval number 2025‐106).

### 2.2 Catheter Ablation and Criteria for Determining Arrhythmia Origin

The PVC exit sites were electro‐anatomically defined as the sites of earliest local activation during ventricular mapping, and were subsequently confirmed by ablation. All mapping and ablation procedures were guided by standard three‐dimensional electro‐anatomical mapping systems, including CARTO (Biosense Webster, Irvine, CA, USA), EnSite (Abbott, Abbott Park, IL, USA), and Rhythmia (Boston Scientific, Marlborough, MA, USA).

The origin was classified as right‐sided origin (RIGHT origin) if the final successful ablation site was located in the right ventricle (RV) or right ventricular outflow tract (RVOT), and as left‐sided origin (LEFT origin) if it was located in the left ventricle (LV) or left ventricular outflow tract (LVOT). Cases originating from the LV summit that demonstrated the earliest activation in the distal coronary sinus were categorized as left‐sided for the purpose of this analysis, because those cases were effectively treated via a left‐sided anatomical approach.

### 2.3 Image Preparation and Evaluation

This study utilized 12‐lead ECGs from 157 patients, comprising 103 cases of RIGHT origin and 54 of LEFT origin. The raw 12‐lead ECG recordings were collected as Joint Photographic Experts Group (JPEG) image files. To ensure data privacy and focus exclusively on waveform analysis, all header information was removed, and the remaining ECG grids were resized to 224 × 224 pixels for input into the model [19].

Model performance was evaluated using a five‐run independent testing approach with StratifiedShuffleSplit (test size = 0.2) across five distinct seeds (0, 2, 4, 6, 8). In this analysis, left‐sided origin was defined as the positive class, and the primary evaluation metrics were positive predictive value (PPV), negative predictive value (NPV), and recall. Furthermore, in this class‐imbalanced setting, both avoiding unnecessary escalation and minimizing missed high‐risk cases are clinically important; therefore, the sum of PPV and NPV (PPV + NPV) was additionally reported as an exploratory composite summary of predictive values, rather than as a threshold‐independent measure of discrimination. Furthermore, to evaluate and compare the overall discriminative performance between the LLM‐based continuous rule‐based score and the CNN‐based model, receiver operating characteristic (ROC) curves were generated, and the areas under the curve (AUCs) were compared.

### 2.4 Baseline CNN‐Based Approach

As a baseline, an end‐to‐end classifier was constructed using the ResNet‐18 architecture. Rather than utilizing standard ImageNet pre‐trained weights, the model was initialized with weights obtained through SimCLR‐based self‐supervised learning specifically pre‐trained on ECG images. This initialization is critical as these weights are optimized for ECG representations, demonstrating high sensitivity to domain‐specific morphological patterns such as QRS duration, ST‐segment changes, and T‐wave morphology. Using these pre‐trained weights as the initial state, supervised fine‐tuning was performed for the PVC origin classification task. The hyperparameters were set as follows: a batch size of 32, 30 epochs, a learning rate of 0.0001, and weight decay of 0.01.

### 2.5 LLM Based One‐Shot Approach (Chat GPT 5.4)

For the LLM based one‐shot inference approach, a single 12‐lead ECG image was provided as input to predict the origin (RIGHT origin vs. LEFT origin) through a single application programming interface call (Figure 1A). Unlike a simple binary classification task, this approach integrated five distinct diagnostic steps into a single prompt (Supporting Information S1: Table 1):

1. Image quality assessment
2. PVC beat identification
3. V1 polarity evaluation
4. Precordial transition analysis
5. Frontal axis evaluation

**Figure 1:**
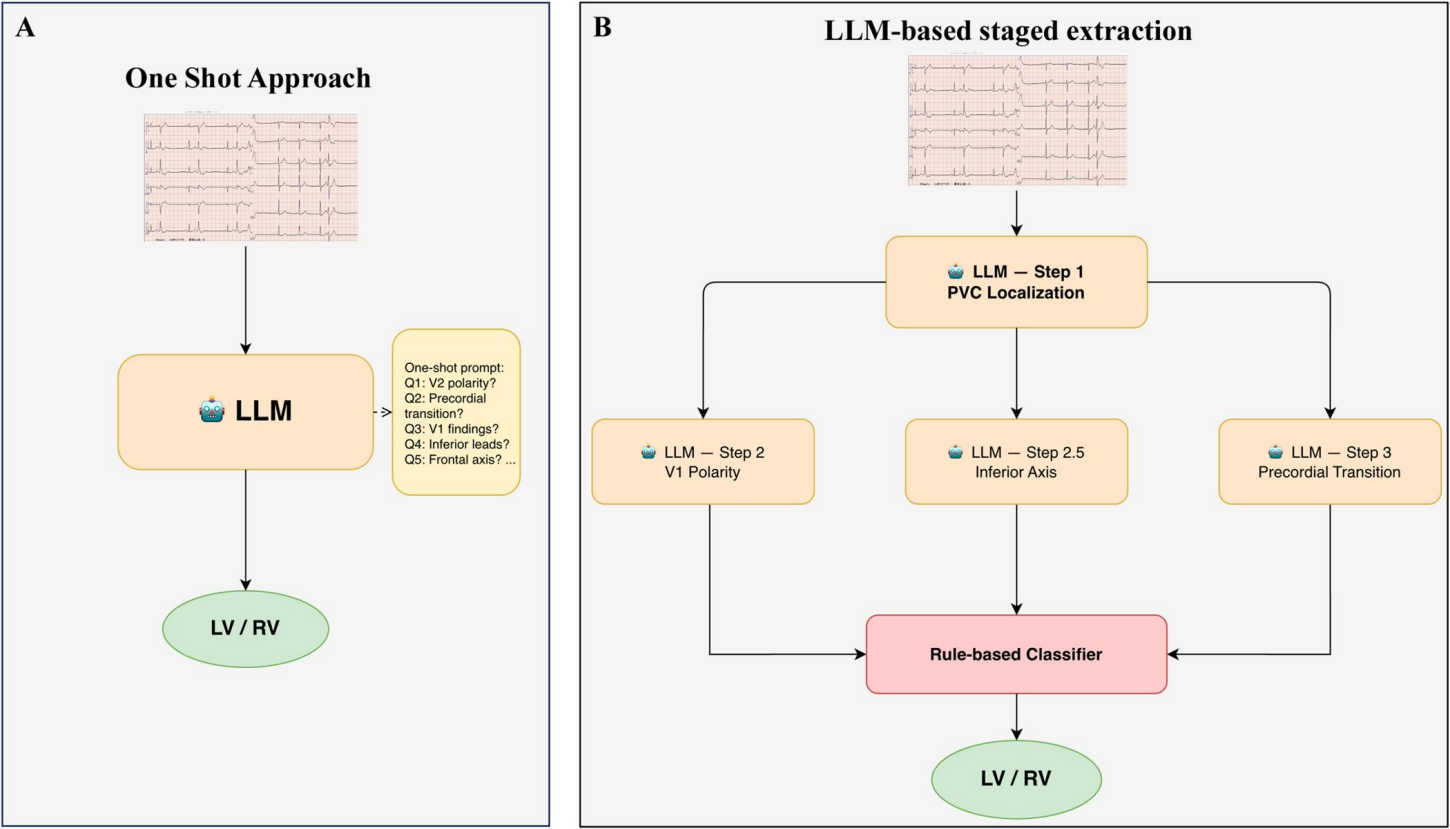
Overview of the LLM‐based frameworks for PVC origin classification. (A) In the one‐shot inference approach, a single 12‐lead ECG image was directly used to classify the PVC origin as right‐ or left‐sided through a single API call. (B) In the staged extraction approach, the diagnostic process was divided into four sequential stages, and the final classification was determined using rule‐based logic based on the outputs of each stage.

**Table 1:** Baseline characteristics.

	RV (*n* = 103)	LV (*n* = 54)	*p* value
Baseline characteristics			
Age	50.9 ± 16.6	63.3 ± 15.2	< 0.001
Male	47	30	0.237
BMI (kg/m^2^)	23.1 ± 3.8	23.8 ± 3.2	0.094
Underlying cardiac disease	12 (11.7)	12 (22.2)	0.08
NICM	5 (4.9)	4 (7.4)	
ICM	6 (5.8)	4 (7.4)	
IVF	3 (2.9)	0 (0)	
CHD (VSD)	1 (1.0)	0 (0)	
Post MVP	0 (0)	1 (1.9)	
Anti arrhythmic drug	25 (24.3)	9 (16.7)	0.27
Na channel blocker	22 (21.4)	7 (13.0)	
Amiodarone	2 (1.9)	1(1.9)	
Bepridil	1 (1.0)	1 (1.9)	
β blocker	57 (55.3)	28 (51.9)	0.68
Laboratory data			
BNP (pg/mL), IQR	38.0 (18.1–123.3)	88.7 (48.4–174.9)	0.007
Creatinine (mg/dL)	0.76 ± 0.18	0.86 ± 0.21	0.11
eGFR (mL/min/1.73m^2^)	73.0 ± 16.4	63.3 ± 17.5	< 0.001
Echocardiography			
LVEF (%)	59.1 ± 11.0	55.0 ± 10.6	0.016
LVDd (mm)	50.5 ± 5.7	51.0 ± 6.1	0.725
Procedure detail			
Arrhythmia type: PVC	98 (95.1)	51 (94.4)	0.84
Arrhythmia type: VT	5 (4.9)	3 (5.6)	
CARTO	76 (73.8)	48 (88.9)	
Ensite	21 (20.4)	1 (1.9)	
Rhythmia	6 (5.8)	5 (9.3)	
Success	93 (90.3)	44 (81.5)	0.90

Abbreviations: BMI, body mass index; BNP, brain natriuretic peptide; CHD, congenital heart disease; ICM, ischemic cardiomyopathy; IVF, idiopathic ventricular fibrillation; LVEF, left ventricular ejection fraction; LVDd, left ventricular diastolic diameter; LV, left ventricle; MVP, mitral valve plasty; NICM, non‐ischemic cardiomyopathy; PVC, premature ventricular contraction; RV, right ventricle; VSD, ventricular septal defect; VT, ventricular tachycardia.

To ensure consistent data extraction, outputs were constrained using Pydantic schemas. The final classification was determined by a majority‐vote logic, defined as the direction supported by at least two of the three primary diagnostic axes.

During the experiments, the prompts were provided to the LLM in Japanese.

### 2.6 LLM‐Based Staged Extraction (Chat GPT 5.4)

To address the limitations of the one‐shot approach, we developed an “LLM‐based staged extraction” framework (Figure 1B), which explicitly decomposes the diagnostic process into four distinct stages (Supporting Information S1: Table 2). In this method, the initial PVC localization is isolated as an independent step, and subsequent modules perform their analysis by referencing the outputs of the preceding stages. The final classification is then determined by a deterministic rule‐based logic.

**Table 2:** Experimental results for each experimental model.

Condition	PPV (Precision)	NPV	Recall	Accuracy	PPV + NPV	AUC
CNN (SSL + ResNet‐18)	0.546 ± 0.058	0.793 ± 0.047	0.600 ± 0.141	0.693 ± 0.038	1.339 ± 0.076	0.712 ± 0.054
LLM one‐shot approach	0.417 ± 0.333	0.682 ± 0.037	0.180 ± 0.117	0.647 ± 0.065	1.099 ± 0.0385	N.A^a^
LLM‐based staged extraction (Baseline setting)	0.633 ± 0.085	0.750 ± 0.023	0.455 ± 0.057	0.719 ± 0.040	1.383 ± 0.104	0.720 ± 0.045
LLM‐based staged extraction (Strict setting)	0.817 ± 0.153	0.727 ± 0.029	0.309 ± 0.093	0.738 ± 0.040	1.544 ± 0.173	0.720 ± 0.045^b^

*Note:* Of the 181 initially screened patients, 24 were excluded according to the pre‐specified criteria (14 lacked baseline ECGs, 4 had pacemaker rhythms, 4 had narrow‐QRS VT, and 2 had multifocal PVCs), resulting in a final study cohort of 157 patients. Supporting Information S2: Figure 1: Patient Eligibility Flow Chart.

^a^ For the one‐shot approach, a full ROC curve could not be constructed because the final output was a binary classification based on majority voting and no continuous confidence score was generated.

^b^ The baseline and strict settings were derived from the same continuous rule‐based score, therefore, they share the same AUC.

The outputs from each stage are converted into numerical features, which are then integrated using a deterministic rule‐based logic implemented in Python. A key advantage of this modular architecture is its ability to mitigate error propagation. If Step 1 indicates uncertainty in PVC localization, this metadata is passed to subsequent stages. By explicitly handling such uncertainties rather than forcing a classification, the system effectively suppresses the cascade of misidentifications that typically leads to diagnostic errors in end‐to‐end models.

Each ECG finding was converted into predefined numeric features and additively integrated into a rule‐based score. The apparent score ranges reflected additive contributions from separate ECG features rather than variable weighting within a single feature. The full scoring rules and criteria for each ECG finding are provided in Supporting Information S1: Table 3. Based on these criteria, features suggestive of a LV origin were assigned negative scores, while features suggestive of an RV origin were assigned positive scores to perform feature extraction. Finally, based on the cumulative score, the site of origin (RIGHT vs. LEFT) was determined using two predefined threshold settings.

### 2.7 Baseline Cut‐Off Setting

A score < 0 was classified as a LEFT origin. This represents a permissive criterion, in which the presence of even minimal LV‐supportive evidence was sufficient to assign an LV origin. Conversely, a score ≥ 0 was classified as a RIGHT origin. This zero‐centered decision boundary inherently arises from the algebraic sign of the cumulative score, where negative values support a LEFT origin and positive values support a RIGHT origin. As such, it serves as a neutral, baseline reference point designed directly into the scoring logic without requiring any additional parameter optimization.

### 2.8 Strict Cut‐Off Setting

In addition to the baseline threshold, we evaluated a stricter threshold (score ≤ −5), determined using the training subset of each split. For each of five random seeds (0, 2, 4, 6, 8), the full dataset was divided into training (80%) and test (20%) subsets using stratified random splitting (scikit‐learn StratifiedShuffleSplit, test_size = 0.2), identical to the procedure used for CNN training. The strict threshold was selected from the training subset as the score that maximized PPV, and model performance was subsequently evaluated on the held‐out test subset. This threshold represents a high‐PPV operating point derived from the training data; rather than reflecting the amplification of a single clinical feature, it strictly identifies cases where multiple independent negative (LV‐supportive) findings overlap. Scores not meeting the above criteria were classified as RIGHT origin.

### 2.9 Statistical Analysis

All performance metrics are reported as mean ± standard deviation or median with interquartile range. Continuous variables were summarized as the median and compared using the Mann–Whitney *U* test. Categorical variables were summarized as proportions and compared using the *χ* ^2^ test. For CNN models, individual ROC curves for each seed and the mean ROC curve were generated. For the LLM‐based staged extraction approach, ROC curves were generated using the continuous rule‐based score before applying the predefined classification thresholds. Because lower scores indicated stronger evidence for a LEFT origin, and LEFT origin was defined as the positive class, the sign‐reversed score (−score) was used as the continuous prediction score for ROC analysis. The baseline cutoff (score < 0) and strict cutoff (score ≤ −5) were treated as operating points on the same continuous score. Therefore, the AUC was identical for the baseline and strict threshold settings. To rigorously compare the diagnostic performance between the CNN and LLM models, a paired DeLong test was performed on the AUCs obtained across the five random seeds. A value of *p* < 0.05 was considered statistically significant unless specified otherwise.

## 3 Results

Among the initial 181 consecutive cases, 24 were excluded based on the following criteria (Supporting Information S2: Figure 1):

1. VT cases without a baseline 12‐lead ECG recording of the index PVC waveform.
2. Pacemaker‐rhythm cases.
3. Verapamil‐sensitive left ventricular tachycardia presenting with a narrow QRS morphology.
4. Cases presenting with multifocal PVC waveforms on the 12‐lead ECG.

Consequently, a total of 157 patients were enrolled in this study (RIGHT origin, *n* = 103; LEFT origin, *n* = 54) (Table 1).

### 3.1 Predictive Performance of the CNN‐Based Approach

The CNN‐based baseline model achieved a PPV of 0.546 ± 0.058 and a negative predictive value NPV of 0.793 ± 0.047 (Table 2). Figure 2 illustrates the ROC curves across five independent seeds. Despite inherent variations across the different seeds, the model demonstrated consistent predictive performance, with area under the curve values ranging from 0.595 to 0.730. Furthermore, a formal statistical comparison using a paired DeLong test was performed to evaluate the diagnostic performance against the LLM‐based framework. Across all five random seeds, the paired DeLong test yielded *p*‐values ranging from 0.264 to 1.000 (*p* = 0.794, 0.490, 0.385, 0.264, and 1.000, respectively), confirming that there was no statistically significant difference in AUC between the CNN and LLM approaches.

**Figure 2:**
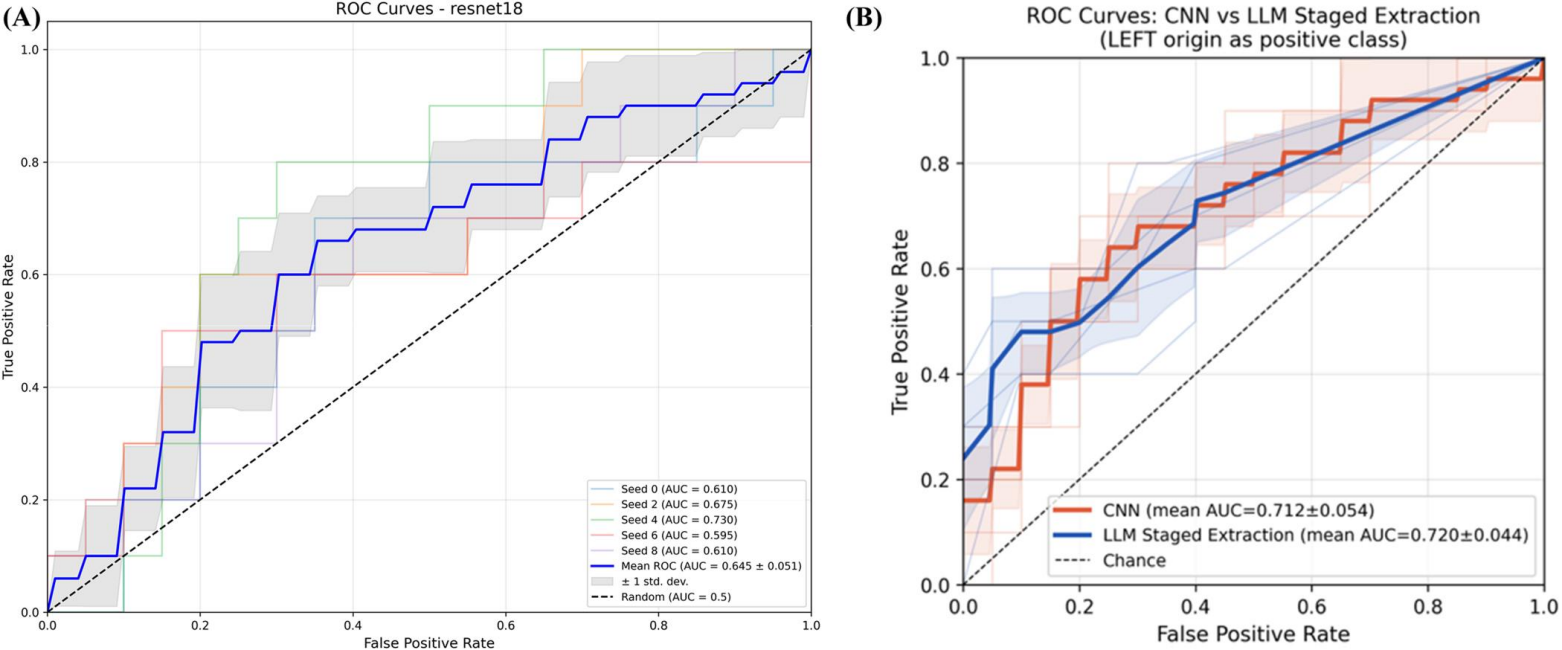
ROC curves for PVC origin classification. (A) ROC curves of the CNN‐based model across five independent seeds. Despite variability across seeds, the model showed consistent predictive performance, with AUC values ranging from 0.595 to 0.730. (B) ROC curves of the LLM‐based staged extraction framework generated by varying the threshold of the continuous rule‐based score. Because lower scores indicated stronger evidence of a LEFT origin, the sign‐reversed score was used for ROC analysis, with LEFT origin defined as the positive class. The staged extraction framework achieved an AUC numerically comparable to that of the CNN‐based model. *Positive: LEFT origin, Negative: RIGHT origin.

### 3.2 Predictive Performance of the LLM‐Based Approach

ROC analysis using the continuous rule‐based score showed that the LLM‐based staged extraction framework achieved an AUC of 0.720 ± 0.045, which was numerically comparable to that of the CNN‐based model (0.712 ± 0.054). The performance metrics for the LLM‐based approaches are also summarized in Table 2. The LLM‐based staged extraction consistently outperformed the LLM one‐shot approach across all evaluated parameters. The baseline and strict thresholds corresponded to different operating points on the same ROC curve. Specifically, the strict threshold, determined using training data, increased PPV but reduced recall, indicating a potential high‐PPV operating point rather than superior overall discrimination for identifying LEFT‐origin PVCs. In contrast, the CNN‐based model yielded the highest results for NPV and recall, suggesting a more robust overall detection capability for the majority class despite the specialized precision of the staged LLM approach. The LLM‐based staged extraction with the strict setting achieved the highest PPV + NPV among the evaluated approaches.

Figure 3 shows the score distribution generated by the rule‐based classifier. The distribution demonstrated a bimodal pattern extending in both the positive and negative directions around 0, with approximately 21% of cases distributed between −5 and 0. In this specific range, the distribution was roughly evenly split (about 50% LEFT origin and 50% RIGHT origin), representing the most challenging diagnostic gray zone. Anatomically, this boundary region predominantly comprised cases located near the septal aspect of the RVOT or the ventricular coronary cusps.

**Figure 3:**
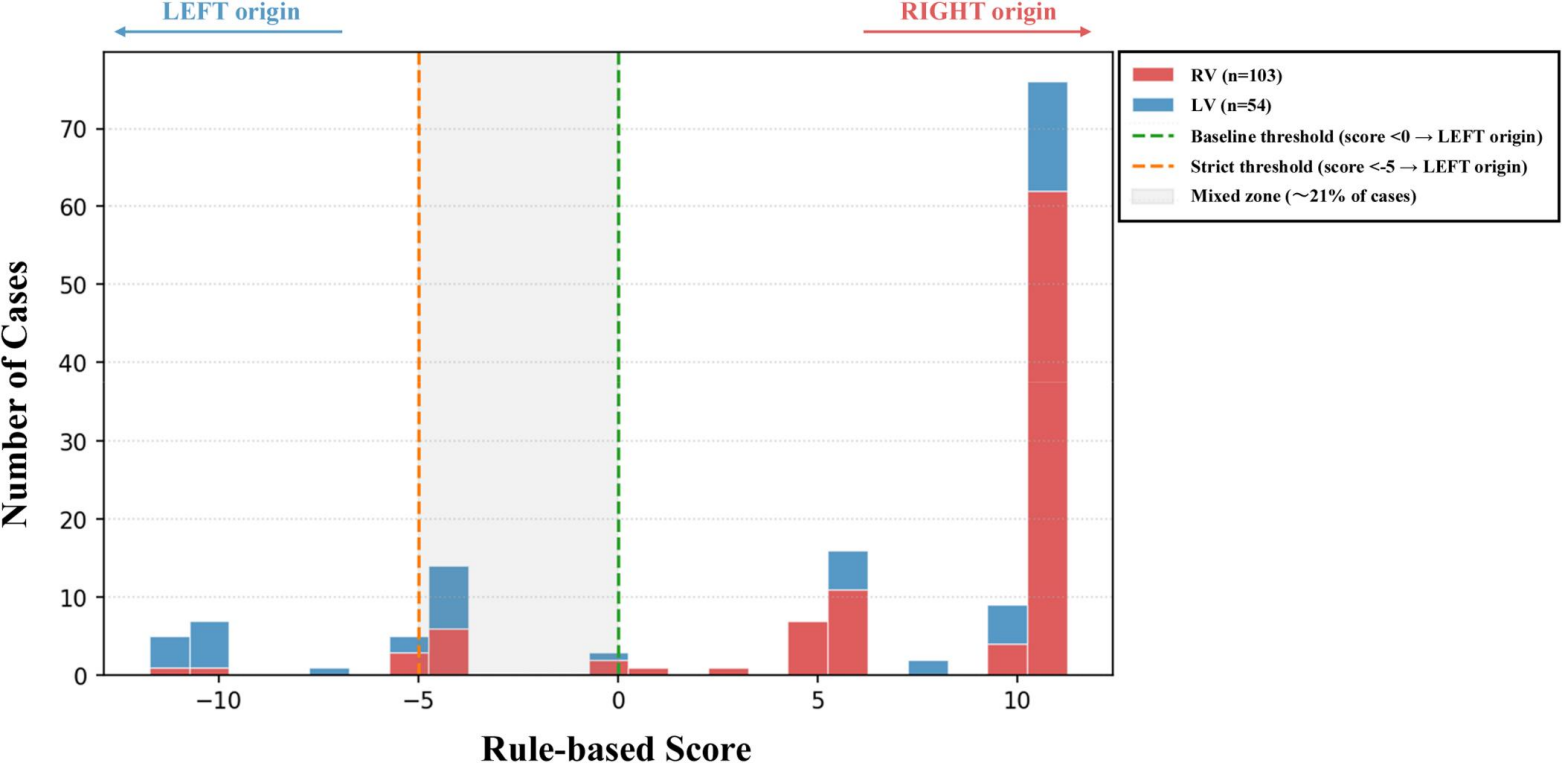
Score distribution by true PVC origin using the rule‐based classifier. The score distribution showed a bimodal pattern centered around 0, with approximately 21% of cases distributed between −5 and 0. Because complete separation was not achieved, a strict threshold was applied to classify this overlapping range as right‐sided rather than left‐sided, thereby improving positive predictive value. However, from a clinical safety perspective, this overlapping range represents a gray zone with a near‐equal split between actual origins and is practically designated as “inconclusive” to prompt mandatory specialist evaluation.

Because complete score separation was not achieved even with the LLM‐based staged extraction approach, applying the strict threshold improved PPV by classifying this overlapping score range (−5 to 0) as RIGHT origin rather than LEFT origin. However, from a practical standpoint, this mathematical classification should not be relied upon blindly; in clinical practice, this gray zone is best managed as “inconclusive,” serving as a safety net that triggers mandatory specialist review.

### 3.3 Representative Case of the LLM‐Based Staged Extraction

In cases where the LLM‐based staged extraction successfully identified the site of origin, Step 1 accurately localized the PVC. Subsequent analyses in Steps 2–4 evaluated the precordial transition and correctly estimated a RIGHT origin based on the transition zone, with the rationale for each stage explicitly provided (Figure 4A and Supporting Information S1: Table 4).

**Figure 4:**
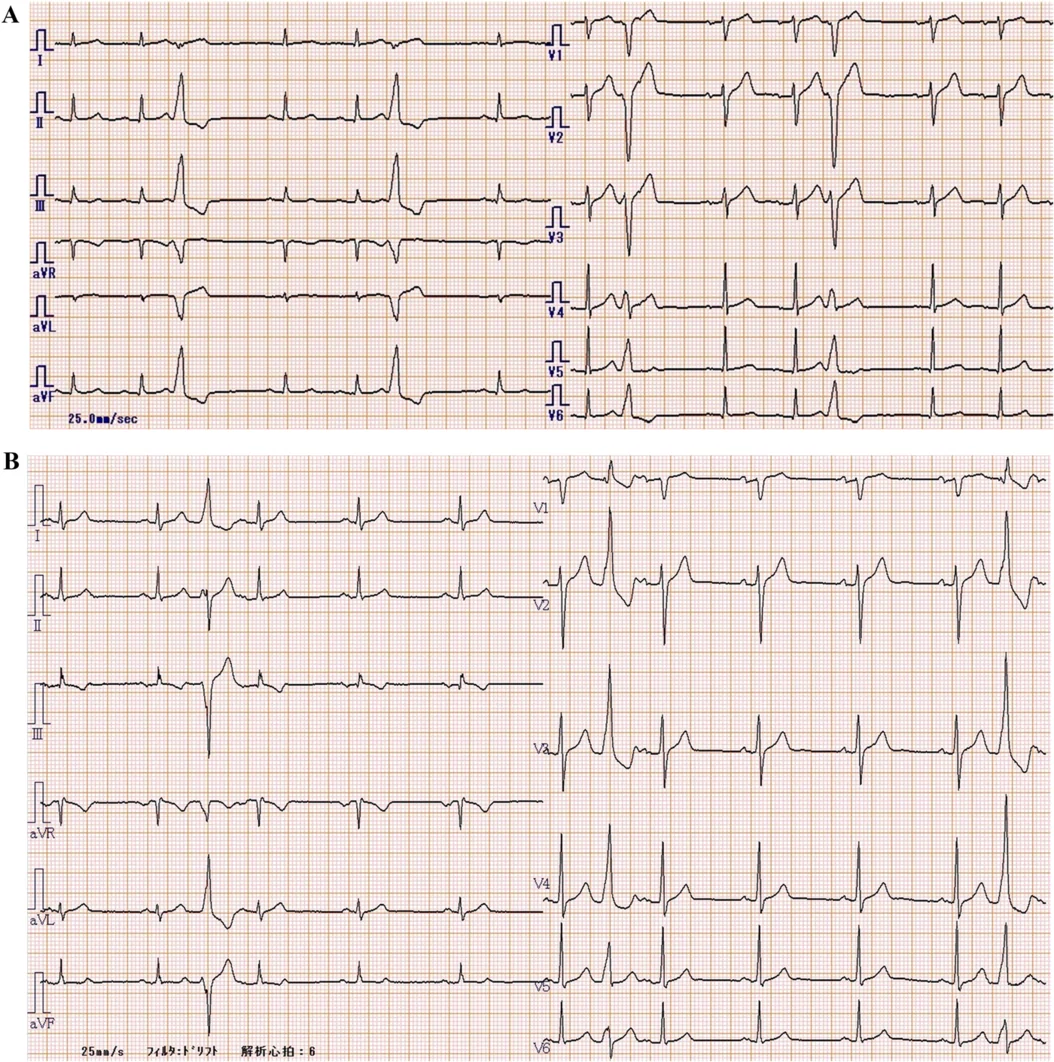
Representative case of the LLM‐based approach. (A) Representative case of successful prediction using the LLM‐based staged extraction approach. In a correctly classified case, staged analysis accurately localized the PVC and identified a RIGHT origin based on precordial transition. The rationale for each step is shown in Supporting Information S1: Table 4. (B) Representative case of failed prediction using the LLM‐based one‐shot approach. In a representative misclassified case, the model estimated a RIGHT origin based on an incorrect interpretation of lead V1 polarity. Manual review indicated that the PVC showed a predominantly positive V1 morphology, whereas the negative‐dominant pattern was present in the baseline sinus rhythm, suggesting that the model may have focused on the sinus rhythm rather than the PVC. The model‐generated rationale is shown in Supporting Information S1: Table 5.

Conversely, in a representative case of misclassification, the model's rationale stated, “Lead V1 of the PVC shows negative dominance (QS/S dominance); v1_polarity supports RIGHT… Since at least two of the three primary axes support a right‐sided origin, the origin is estimated as RIGHT.” However, manual review revealed that the actual PVC morphology demonstrated a positive concordant pattern with a predominantly positive V1 component. In contrast, the baseline sinus rhythm in lead V1 exhibited a negative‐dominant (QS/S‐like) morphology. This discrepancy suggests that the LLM may have erroneously focused on sinus rhythm instead of the PVC during its diagnostic process (Figure 4B and Supporting Information S1: Table 5).

## 4 Discussion

### 4.1 Major Findings

The major findings of this study were as follows:

1. The LLM‐based ECG prediction model demonstrated the basic feasibility of classifying PVC origins as right‐ or left‐ventricular, achieving this binary differentiation without the need for task‐specific, large‐scale training datasets or model retraining.

2. The LLM framework enabled explicit representation of the criteria underlying classification, thereby improving interpretability compared with conventional CNN‐based approaches.

3. LLM‐based staged extraction with explicit intermediate outputs and deterministic integration achieved a more favorable high‐PPV operating point than the one‐shot approach.

### 4.2 Clinical Utility of an LLM‐Based Prediction Model Using ECG Images

AI‐based ECG analysis has advanced rapidly, and disease‐specific models for PVC localization have also been developed [12, 20]. However, CNN‐based models remain vulnerable to overfitting when training data are limited, and their generalizability may depend substantially on the quantity and quality of available ECG images. Although we previously showed that self‐supervised learning can improve CNN performance in small‐image settings, the challenge of robust generalization across external datasets and different recording environments remains [19].

Against this background, the present study evaluated an alternative strategy based on LLMs for ECG image interpretation. Specifically, our results demonstrated that the staged extraction approach, particularly when combined with a strict threshold, achieved a superior high‐PPV operating point without requiring task‐specific retraining. Because LLMs leverage large‐scale pretraining to acquire broad visual and linguistic representations, they may offer a distinct diagnostic framework for arrhythmia applications, where annotated disease‐specific data sets are often limited.

A further strength of this study is that the model directly analyzed routine 12‐lead ECG image data rather than digital waveform signals. This image‐based approach may be advantageous from a practical standpoint, as it does not require rigid signal preprocessing or strict input standardization. Such flexibility may ultimately improve adaptability across heterogeneous clinical environments and may facilitate broader implementation, including at centers without advanced AI development infrastructure.

Recent literature highlights the expanding role of LLMs in cardiology, primarily demonstrating robust capabilities in image‐based interpretation for echocardiography and coronary angiography. However, applying LLMs to 12‐lead ECG analysis introduces unique challenges. Recently, Gürses et al. evaluated the diagnostic accuracy of ChatGPT for PVC localization by feeding language‐based text prompts containing extracted ECG parameters (such as QRS polarity and transition zones) [15]. While such text‐based approaches eliminate the need for image processing, they heavily rely on human operators to accurately measure and input discrete parameters first. In contrast, our study explored the feasibility of a multimodal LLM directly extracting visual geometric features from raw ECG images, which represented a more seamless and automated clinical workflow, albeit with a slight trade‐off in initial binary accuracy.

### 4.3 Interpretability as a Key Advantage of the LLM‐Based Framework

In conventional CNN‐based models, techniques such as Grad‐CAM can highlight the image regions that influence prediction; however, they do not fully explain why those features led to a particular diagnostic conclusion. As a result, their explanatory value remains limited when clinicians must judge whether a model output is trustworthy in an individual case.

By contrast, the LLM‐based framework used in this study preserved stepwise natural‐language reasoning as intermediate outputs, allowing the diagnostic process to be traced in a manner aligned with established ECG interpretation. This feature substantially enhances “interpretability” by making the basis of classification more explicit. In particular, the ability to map the reasoning process to familiar electrophysiologic features, such as V1 polarity and precordial transition, may improve clinical transparency and acceptance.

When compared to task‐specific deep learning networks, however, the performance of general‐purpose LLMs shows a noticeable baseline gap. For instance, high‐precision CNN algorithms, such as those developed by Chang et al. [21], achieve remarkable accuracy in localizing idiopathic ventricular arrhythmias by utilizing raw digital signal datasets and extensive task‐specific training. This also explains the substantial difference in accuracy between our current LLM model and the deep learning‐based semantic segmentation model reported by Kujime et al. [13]. The model by Kujime et al. achieved high‐precision localization because it was meticulously trained on thousands of digitized ECG leads specifically to segment and analyze pixel‐level QRS features. Our zero‐shot or point‐based LLM framework does not undergo such large‐scale, task‐specific weight tuning. Instead, it relies on general visual reasoning, which inherently limits its resolution for subtle morphological nuances but offers the distinct advantage of high interpretability without requiring extensive computational retraining.

At the same time, caution remains necessary. LLM‐generated explanations should not automatically be assumed to represent the model's true internal reasoning with complete fidelity. Nevertheless, the ability to externalize and inspect the path to the final prediction represents an important conceptual and practical advantage over more opaque end‐to‐end models.

### 4.4 Importance of Staged Extraction in LLM‐Based Prediction

The present findings suggest that, for ECG image interpretation by LLMs, a staged extraction approach may be more effective than one‐shot end‐to‐end prompting [22]. In the one‐shot approach, PVC identification and morphologic interpretation are handled within a single inference process, making the final classification more susceptible to upstream recognition errors. In contrast, the staged extraction approach separates PVC localization from subsequent feature evaluation, thereby reducing error propagation and yielding a more controlled diagnostic pipeline.

This observation has broader implications. For tasks such as PVC localization, the final diagnosis is not a single visual judgment but the result of several sequential interpretive steps, including beat identification, assessment of V1 polarity, evaluation of precordial transition, and axis determination. In such settings, explicit decomposition of the task, coupled with deterministic integration, appears better suited to preserve interpretability and improve diagnostic precision.

Importantly, however, the comparison between staged extraction and the one‐shot approach should be interpreted as a comparison of overall pipelines rather than of task decomposition alone. Prompt structure, explicit intermediate outputs, and rule‐based final integration all differed simultaneously, and the relative contribution of each component cannot be determined from the present data.

Finally, the score distribution offers an additional insight. Although most cases clustered at clearly positive or clearly negative values, approximately 21% fell within the intermediate range between −5 and 0, indicating persistent overlap between typical and borderline cases even under staged extraction. The improved PPV observed with the strict setting likely reflects the use of a higher threshold for LEFT‐origin classification, effectively shifting this ambiguous range toward RIGHT‐origin assignment.

Electrophysiologically, this overlap reflects the anatomical proximity of the RVOT and LVOT. In this boundary region, two major clinical factors heavily confound ECG morphology: (1) individual variations in cardiac rotation, which shift the precordial QRS transition zone, and (2) lead placement variability, where minor electrode shifts (especially V1 and V2) alter the apparent R/S ratio. Consequently, cases near the septal RVOT or coronary cusps produce highly overlapping QRS patterns, making perfect binary separation on a surface ECG inherently difficult.

Therefore, this overlap represents a known clinical limitation driven by cardiac axis and recording variability, rather than an algorithmic flaw. To ensure clinical safety, this intermediate gray zone should practically be treated as “inconclusive,” serving as an automatic trigger for mandatory evaluation by an electrophysiologist. Defining this range as a “clinical gray zone” provides realistic and transparent insight, demonstrating how AI can identify its own diagnostic boundaries to complement human expertise.

### 4.5 Comparison of Our Approach With Manual Review

Our model's performance should be interpreted in the context of manual human ECG review. While expert electrophysiologists can achieve high accuracy in differentiating RV vs. LV origins, the necessary 12‐lead ECG interpretation requires complex, multi‐step algorithms that remain universally challenging and time‐consuming for non‐electrophysiologists. Consequently, reliable manual interpretation presents a significant hurdle in general settings. Given these clinical challenges, the baseline performance demonstrated by our LLM‐based model highlights its utility in bridging this diagnostic gap. This suggests that while LLMs cannot currently replace specialized CNNs or expert electrophysiologists, they hold substantial potential as a rapid, explainable screening tool for general practitioners.

## 5 Clinical Implications

Localization of the origin of PVCs is generally a well‐established skill for electrophysiologists, and in typical cases, it can be performed with relative ease. However, for non‐electrophysiologists, detailed morphological interpretation of 12‐lead electrocardiograms is not always intuitive, and accurate estimation of the PVC origin may be challenging. Importantly, PVCs arising from the LV differ from those originating from the RVOT in that they are more frequently associated with underlying coronary artery disease or cardiomyopathy and may therefore carry greater clinical significance.

Accordingly, even a coarse classification into right‐sided versus left‐sided origin may provide clinically meaningful information for non‐specialists. The LLM‐based framework evaluated here features a structured pathway for interpreting ECG images, offering a transparent, clear diagnostic process to differentiate left‐sided from right‐sided origins. Although this method warrants future prospective validation before clinical implementation by non‐specialists, this approach could potentially serve as a preliminary, supportive screening tool, provided that any automated outputs are subject to manual review by expert specialists before clinical decision‐making.

## 6 Limitations

This study has several limitations. First, this was a single‐center retrospective study with a relatively small sample size, and it completely lacked prospective external validation, which inherently limits the definitive generalizability of our findings to other clinical settings. Second, the classification task was simplified to right‐versus left‐sided origin, and more detailed anatomical localization, such as differentiation within the outflow tract or summit region, was not evaluated. Incorporating precise, segment‐specific ablation sites will be an important consideration for the design of future studies to further refine the model's diagnostic resolution. Third, the ECGs were analyzed as static image data rather than raw digital waveform signals, and image quality or layout differences may have influenced model performance. Fourth, the LLM‐based staged extraction and one‐shot approaches differed in multiple design elements, including prompt structure, task decomposition, and deterministic integration; therefore, the contribution of each individual component could not be isolated. Fifth, our binary RV/LV classification is inherently coarse. Complex origins like the LV summit require entirely different procedural planning and vascular access, making expert manual review indispensable. Furthermore, approximately 21% of cases fell into an intermediate score range with a near‐equal split between actual RV and LV origins. To maintain clinical safety, this gray zone should be treated as “inconclusive” in practice, serving as an automatic trigger for mandatory evaluation by an electrophysiologist rather than relying on the automated output. Additionally, the strict threshold of −5 was determined using training data and not prospectively validated in an independent external cohort. Accordingly, the reported performance under this setting should be interpreted with caution, and prospective or external validation is warranted before clinical application. Finally, although the LLM‐generated intermediate reasoning improved interpretability, whether these outputs faithfully reflect the model's true internal decision process requires further validation.

## 7 Conclusions

LLM‐based staged extraction demonstrated the potential to achieve binary left‐versus‐right PVC origin localization from 12‐lead ECG images while providing a traceable stepwise diagnostic process. The continuous rule‐based score showed discrimination numerically comparable to the CNN‐based model, and the strict threshold—determined using training data—identified a potential high‐PPV operating point that requires prospective external validation.

## Author Contributions

K.M., Y.F., and H.M. provided the study concept and design, data analysis, and drafted the manuscript. H.S., M.N., W.S., T.N., N.T., D.K., K.M., and W.Y. contributed to the data analysis and interpretation. Y.I. and R.K. reviewed and revised the manuscript draft.

## Disclosure

H.M. received lecture fees from Biosense Webster Japan and Boston Scientific Japan. Our department received grant support from Boston Scientific Japan and Abbott Medical Japan.

## Ethics Statement

The study protocol was approved by the hospital's institutional review board (IRB number; 2025‐106).

## Supporting information


Supporting File 1

Supporting File 2

## Data Availability

The data that support the findings of this study are available from the corresponding author upon reasonable request.
