# Supplementary material for: Large Language Model‐Based Localization of Premature Ventricular Contraction Origins: A Retrospective Diagnostic Accuracy Study
Source: J Cardiovasc Electrophysiol. 2026 Aug 1;37(11):2587–97. doi: 10.1111/jce.70464 (PMC13768791; doi:10.1111/jce.70464)
Supplement: Supplementary file 1 [file JCE-37-2587-s002.docx]

**Supplemental Table 1:** **Prompt Instructions for the LLM based One-shot Approach**

**Role:**

You are an expert in electrocardiogram (ECG) interpretation. Based on the provided 12-lead ECG images, estimate the site of origin for non-reentrant ventricular arrhythmias (PVC/VT) as a binary classification: RIGHT (right-sided origin) or LEFT (left-sided origin). The analysis should target the specific beat where the arrhythmia occurs (e.g., the beat indicated by a red frame).

**#Important Rule: Do Not Quantify Unless Measurable (Mandatory)**

-Definitive quantitative measurements (e.g., QRS duration in ms, R/S ratios) are permitted ONLY when the following criteria are met:

(a) Calibration grids (small squares) are clearly visible.

(b) The onset and offset of the waveform (QRS start/end, R-peak, S-wave termination) are distinctly identifiable.

-If these conditions are not met, do not provide definitive values. Instead, specify the "measurement_basis" as either "measured," "estimated", or "not_assessable."

**# Step 0: Quality and Measurability Assessment**

Initially, evaluate the following parameters:

- grid_visible: Are the grid lines discernible?
- leads_identifiable: Are at least the 12 standard leads (V1–V6 and I/II/III) identifiable?
- pvc_beat_identifiable: Is the target PVC/VT beat clearly identified (e.g., by a red frame or distinct morphology)?
- waveform_readable: Is the waveform free from significant distortion or clipping?

Lower the confidence score (e.g., 0.2–0.6) if these qualities are insufficient.

**# Step 1: Beat Identification**

- Confirm if the alphanumeric lead labels are recognizable
- Determine if a QRS duration exceeding 120 ms is observed.

**# Step 2: Validation of Target Beat (PVC/VT Characteristics)**

- Evaluate the following as present, suspected, or unknown
- wide_qrs, abnormal_morphology, and fixed_coupling

**# Step 3: Core Findings for Origin Estimation (Three Primary Axes)**

- V1 Polarity: (support_RIGHT / support_LEFT / inconclusive)
- Precordial Transition: (Early → LEFT / Late → RIGHT)
- Frontal Axis: Orientation based on leads I, II, III, aVF, aVL, and aVR

**# Step 4: Supplementary Markers**

Assess for additional features:

- rbbb_like_pattern, lbbb_like_pattern, inferior_leads_positive, and precordial_concordance.

**#** **Final Decision Logic**

The final determination of RIGHT or LEFT is based on a majority-vote logic: the direction supported by at least two of the three primary axes. If all primary axes are inconclusive, the confidence score must be reduced to 0.2–0.35.

＊The instructions provided to the LLM during the experimental phase were implemented and validated in Japanese, as described above.

**Supplemental Table 2:** **Prompt Instructions for LLM-based staged extraction**

**Step 1: PVC Localization –**

Identifies PVC beats in leads where they are easily discernible (e.g., II, III, aVF, V2, or V3) and evaluates the feasibility of mapping these findings to lead V1.

**Step 2: V1 Polarity –**

Evaluates the primary polarity of the representative PVC beat identified in Step 1 as RIGHT, LEFT, or UNCERTAIN.

**Step 2.5: Inferior Axis –**

Extracts axis hints (inferior or superior) based on the primary polarity in leads II, III, and aVF.

**Step 3: Precordial Transition –**

Determines the transition zone (early, intermediate, or late) based on the primary polarities in leads V2, V3, and V4 (Figure 1a).

＊The instructions provided to the LLM during the experimental phase were implemented and validated in Japanese, as described above.

**Supplemental Table 3: Scoring Rules and Diagnostic Criteria for PVC Origin Localization**

| **Feature** | **Condition** | **Score** |
| --- | --- | --- |
| **Transition zone** | **Early Transition** | **-3** |
| **Transition lead** | **≦V2** | **-2** |
| **V2 polarity** | **Positive** | **-3** |
| **V1 overall judgment** | **Suggestive of LV origin** | **-2** |
| **V1 polarity** | **Positive / RBBB-like** | **-1** |
| **V2 polarity** | **Non-positive** | **+2** |
| **Transition zone** | **Late Transition** | **+3** |
| **Transition lead** | **≧V4** | **+2** |
| **V1 overall judgment** | **Suggestive of RV origin** | **+2** |
| **V1 polarity** | **Negative / LBBB-like** | **+1** |
| **Inferior axis** | **Positive deflections in all inferior leads** | **+0.5** |

**Supplemental Table 4:** **Representative cases illustrating the interpretability of LLM-Based Staged Extraction for ECG interpretation**

**# Step 1: PVC Localization**

- Reasoning: A repetitive sequence of wide QRS complexes, distinctly different from the sinus rhythm, was observed in leads II, III, and aVF. Due to the morphological monomorphism and recurrence, the pattern was classified as "repetitive." Lead III was selected as the primary anchor for its high signal-to-noise ratio. Consistent QRS abnormalities were synchronized across leads aVF, V2, and V3. The identification was further validated by the unambiguous presence of corresponding abnormal complexes in lead V1.
- Output Parameters:

・pvc_pattern_mode: Repetitive

・anchor_lead: III

・sync_to_v1_trustworthy: True

・confidence: High

**# Step 2: V1 Polarity**

- Reasoning: The representative PVC complex in lead V1 is characterized by a predominantly negative polarity, featuring a deep S-wave with an overall LBBB-like morphology. Based on these findings in the horizontal plane (specifically lead V1), the electrical activation pattern is highly suggestive of a right-sided origin.
- Output Parameters:

・v1_dominant_polarity: Negative

・v1_pattern_hint: LBBB-like

・predicted_origin_from_v1: RIGHT

**# Step 2.5: Inferior Axis:**

- Reasoning: The dominant QRS deflection is upward in leads II, III, and aVF. Each inferior lead demonstrates a broad and predominant R wave, indicating 3/3 positivity in the inferior leads. Therefore, the axis hint is classified as inferior.
- Output Parameters:

　・inferior_positive_count = 3

・axis_hint = inferior

**# Step 3: Precordial Transition:**

- Reasoning: In lead V2, an R wave is present; however, the deep S wave is dominant, resulting in an overall negative polarity. A similar pattern is observed in lead V3. In lead V4, the dominant QRS deflection becomes predominantly positive for the first time, indicating that the precordial transition occurs at V4. Accordingly, the transition group is classified as late.
- Output Parameters:

・v2_polarity = negative

・v3_polarity = negative

・v4_polarity = positive

・transition_lead = V4

・transition_group = late

**#** **Rule-based integration:**

A non-positive V2, late precordial transition, and negative V1 collectively yield a strongly positive cumulative score, supporting a RIGHT origin.

＊The examples shown above represent English translations of outputs originally generated in Japanese.

**Supplemental Table 5: Representative Cases of Failed Predictions Using the LLM-Based One-Shot Approach**

- **Reasoning:**

In the target beat, lead V1 showed a predominantly negative morphology (QS/S-like), and therefore v1_polarity was interpreted as support_RIGHT. Across the precordial leads, predominant negativity persisted from V1 to V3, indicating a delayed transition; accordingly, precordial_transition was also interpreted as support_RIGHT. In the limb leads, lead I was predominantly positive, and thus frontal_axis_hint was likewise interpreted as support_RIGHT. Because at least two of the three major axes supported a right-sided origin, the beat was estimated to originate from the RIGHT side.

＊The examples shown above represent English translations of outputs originally generated in Japanese.
